# Depression and anxiety behaviour in a rat model of chronic migraine

**DOI:** 10.1186/s10194-017-0736-z

**Published:** 2017-02-21

**Authors:** Mingjie Zhang, Yufei Liu, Mangsuo Zhao, Wenjing Tang, Xiaolin Wang, Zhao Dong, Shengyuan Yu

**Affiliations:** 1 Department of Neurology, Chinese PLA General Hospital, 28 Fuxing Road, Beijing, 100853 People’s Republic of China; 2 Department of Neurology, Tianjin Third Center Hospital, Tianjin, 300170 People’s Republic of China; 3 Department of Neurology, Yuquan Hospital, Medical Center, Tsinghua University, Beijing, 100049 People’s Republic of China

**Keywords:** Chronic migraine, Depression, Anxiety, Scale

## Abstract

**Background:**

Epidemiological and clinical studies have demonstrated comorbidity between migraine and affective disorders. However, it is unclear whether chronic migraine can lead to affective disorders in other animals.

**Methods:**

A classical chronic migraine rat model (repeated dura mater inflammatory soup [IS] infusion) was used to evaluate depression and anxiety behaviour via weight, sucrose preference test, open field test and elevated plus maze test.

**Results:**

We found that sucrose preference, locomotor and rearing behaviours, inner zoon distance percent, open-arm entries percent and serotonin and dopamine levels in the prefrontal cortex decreased significantly in the IS group compared with those in the control group; co-administration of low-dose amitriptyline ameliorated these deficits. However, no differences in weight, inner zone time percent, or open-arm time percent between the IS and control groups. These results were used to create new depression and anxiety scales to comprehensively assess and evaluate the degree of affective disorders in rats. Most of chronic migraine animals showed depression and anxiety like behaviors but a few didn’t.

**Conclusions:**

Most of the chronic migraine rats were present depression and anxiety like behaviors. The new scales we created are expected to use in the future studies to find out the potential mechanism of affective disorders’ ﻿comorbidity.

## Background

Epidemiological and clinical studies have demonstrated that primary headache, particularly migraine, has a bidirectional relationship with depression and anxiety [1–5]. Patients with depression have a more than three-fold relative risk of developing migraine compared with non-depressed patients. Similarly, migraineurs have a more than three-fold relative risk of developing depression compared with patients without migraine [6]. More patients with depression and anxiety suffer chronic daily headache than episodic headache, particularly those with a transformed migraine [7]. Comorbidities of depression and anxiety may increase headache frequency in migraineurs [8]. We reported previously that animals with depression (using olfactory bulbectomy or unpredictable chronic mild stress models) elicited severe nociceptive behaviours and had increased plasma levels of substance P [9, 10]. However, it is unknown whether chronic migraine can lead to affective disorders.

Several chronic migraine animal models have been proposed [11, 12]. Repeated inflammatory soup (IS) infusion on the dura mater as meningeal nociception is one of the most widely used models, as it mimics neurogenic inflammation of migraine pathophysiology. Repeated delivery of IS through a pre-implanted cannula daily induces a short-lasting (about 3-6 h) decrease and a long-lasting (>3 weeks) decrease in the facial withdrawal threshold [13]. The animals also exhibit decreased routine physical activity, increased resting behaviour, and specific ipsilateral facial grooming behaviours after IS infusion [11]. In the present study, we used this animal model to study the effects of chronic migraine on affective disorders.

The Hamilton Depression Scale, Beck Depression Inventory, and Self-rated Depression Scale are used in the clinic to diagnose and evaluate the degree of depression. Recently, stagnation was used as a scale to assess the psychometric properties in the patients with chronic migraine and medication overused headache [14, 15]. The Hamilton Anxiety Scale and Self-Rating Anxiety Scale are used to evaluate the degree of anxiety. However, it is very difficult to evaluate an affective disorder in animals. Anhedonia is a core symptom of depression and is frequently tested using the sucrose preference test [16]. The open field (OF) test examines exploratory behaviour and tension state in new surroundings, and has been used as a depression and anxiety test [17]. Travel distance is representative of locomotor behaviour, and the number of times the animal stands on its hind legs represents rearing behaviour. This behaviour is similar to psychomotor retardation (decreased motor activity) on the Hamilton Depression Scale. Weight change is also a clinical feature of depression [18]. If a subject loses >2 kg in 3 d, they gain 2 points on the Hamilton Depression Scale; therefore, weight is another index parameter. Researchers use these tests to evaluate the success of establishing depression in an animal model, such as in an unpredictable chronic mild stress model [16]. Depression like behavior was also found in other animal models, but it may be slight and not easily to be discovered. An animal scale is needed to integrate all indices to evaluate the degree of depression and anxiety.

In this study, we used a rat model of chronic migraine to evaluate depression and anxiety behaviours in rats, and created scales for these affective disorders.

## Methods

### Ethical concerns

The experimental procedures were approved by the Committee on Animal Use for Research and Education of the Laboratory Animals Centre, General Hospital of Chinese People’s Liberation Army (Beijing, PR China), and were consistent with the ethical guidelines recommended by the International Association for the Study of Pain in conscious animals [19]. Efforts were made to minimise animal suffering.

### Habituation

The experiments were conducted in male Wistar rats (weight, 180–200 g) purchased from the Laboratory Animal Centre, Academy of Military Medical Science of People’s Liberation Army. All rats were housed separately at a constant temperature (22–25 °C).

### Animal grouping

The IS contained 0.2 mM prostaglandin E_2_ and 2 mM each of histamine, serotonin, and bradykinin. The rats were divided into 4 groups: control (CON; *n* = 10); IS (*n* = 10; 1 rat died from intestinal paralysis); IS + amitriptyline (AMI) (*n* = 10); and AMI (*n* = 10). After recovery from the cannula implantation surgery, facial withdraw thresholds were recorded; the sucrose preference, OF, and elevated plus maze (EPM) tests were performed to obtain baseline measurements. We delivered 2 μL IS to the rats in the IS and the IS + AMI groups, and the same volume of saline to the rats in the AMI and CON groups through the cannula using a micro-injector for 21 d. AMI was dissolved in water and given to the rats in the IS + AMI and AMI groups (5 mg/kg, intragastric [i.g.]) [20]. The same volume of water was given to the rats in the IS and CON groups for 21 d. Pain thresholds were tested every day during the 21-d experiment, and weight and sucrose preference was measured and tested every week. The OF and EPM tests were carried out after 21 d.

### Surgical procedures

A cannula was implanted as described previously by Su et al. [21]. An absolute diet was given to the rats the day before the surgery to prevent abdominal distention. The rats were anesthetised with 10% chloral hydrate (4 mL/kg, intraperitoneal [i.p.]) and placed in a stereotactic frame. A horizontal incision was made in the head and all connective tissues were removed with 3% hydrogen peroxide to expose the bregma. A drill was used for a 1-mm-diameter craniotomy (+1.5 mm after the bregma, +1.5 mm lateral), so as not to destroy the dura. A plastic cannula, with a stainless steel needle extending 1 mm from the bottom, was inserted into the hole and fixed with the help of 502 glue. A matched cap was used to close the cannula to prevent clogging. We used dental cement for further fixation, and sutured the incision. All rats received prophylactic treatment with an antibiotic (cefdinir 10 mg/kg, i.g.) for 3 d after surgery. Experiments were conducted 4 d after the surgery.

### Pain threshold examination

Baseline pressure thresholds were obtained with von Frey filaments using the manufacturer’s recommended force values (15, 10, 8, 6, 4, and 2 g), applied perpendicularly to the periorbital region of the rats. A positive response on the von Frey test was documented when the rat stroked its face with the ipsilateral forepaw, and the head recoiled quickly toward the side away from the stimulus, or vocalisation [13]. Facial allodynia was assessed via von Frey testing and calculated as the 50% positive response threshold.

### Sucrose preference test

The sucrose preference test was performed before (baseline) and weekly after IS administration. At the start of the experiment, the animals were trained to drink a 1% sucrose solution by exposing them to sucrose instead of water for 48 h. Then, the rats received a series of sucrose preference tests, preceded by 22 h of food and water deprivation. Each animal was presented simultaneously with two weighed bottles: one contained a sucrose solution (1%) and the other contained water. The two bottles were reweighed 1 h later, and the percent preference for sucrose consumption was calculated. Sucrose preference (%) = sucrose solution consumption/(sucrose solution consumption + water consumption) × 100.

### OF test

The OF test was designed to analyse locomotor and rearing behaviours of rats. The apparatus comprised a circular black base (90-cm diameter) surrounded by black walls (50 cm), as described previously [22]. The inner space was a 60-cm diameter circle; the outside space was annular and 30-cm wide outside the inner space. Illumination was provided by a 40 W bulb. On the day before (Day 0) and 15 d after the olfactory bulbectomy surgery, all rats were tested in the OF for 5 min. Total distance, inner zone distance and inner zone time were recorded by a computerized video tracking system (Ethovision 2.0; Noldus, Wagenigen, The Netherlands). Rearing behaviour (number) from the video was determined by an experimenter. The OF was wiped with a 5% alcohol solution between each test to remove olfactory cues. The percentage of inner zone time (IT%) was calculated as the time in the inner zone/300 s × 100. The inner zone distance percentage (ID%) was calculated as the inner zone distance/total distance × 100.

### EPM test

The EPM test was designed to measure anxiety-like responses and was conducted in a 4-arm maze elevated 70 cm above the floor. The 2 closed arms had 22-cm-high dark walls and the 2 open arms had 0.5-cm-high edges. The angle between the arms was 90°. Illumination was provided by a 40 W bulb. The rats were placed in the centre of the apparatus facing a closed arm for 5 min. The percent of time spent in the open arm, the number of open arm entries, and the total number of arm entries were recorded by a camera mounted 1.3 m above the maze, and analysed using a computerised video tracking system (Ethovision 2.0; Noldus). Time in the open arm, time in the closed arm, open arm entries, and closed arm entries were all recorded by the computerised video tracking system. Open arm time percent (OT%) was calculated as time in the open arm/300 s × 100. Open arm entries percent (OE%) was calculated as entries into the open arm/(entries into the open arm + entries into the closed arm) × 100. These data were analysed by an experimenter blinded to the experimental treatments.

### Standard depression and anxiety disorder scales

Loss of interest scores 4 points, slow movement scores 4 points, and weight loss scores 2 points on the Hamilton Depression Rating Scale. We set up a new standard scale using a highest sucrose preference score of 10 points, and a highest OF test score of 10 points (equal for locomotor and rearing behaviours). The highest score for weight change was 5 points. As anxiety indices were based on the same principle (i.e., fear of a new environment), they were given equal scores of 7 points.

The range of normal values was determined by the CON group results, as animals in this group were not exposed to pain, and they were less likely to be affected by mental disorders. The range was >5% or <5%. The CON group indices were normally distributed. High scores indicated normal behaviour and low scores indicated depression or anxiety for indices other than weight. Overweight and underweight are responses to depression; therefore, we used normal weight as the highest score, and overweight and underweight scored fewer points. The specific scale systems are shown below in Results section “Scales of depression and anxiety disorders in rats”.

### Biochemical analysis of the prefrontal cortex

The rats were sacrificed the day after the depression and anxiety tests. Their skulls were removed, and the prefrontal cortex was dissected and stored at −80 °C for biochemical analyses. Serotonin (5-HT) and dopamine levels were determined in the prefrontal cortex using enzyme linked immunosorbent assay (ELISA) kits (Nanjing Jiancheng Bioengineering Institute, China), according to the manufacturer’s instructions (R&D Systems, Minneapolis, MN, USA). Four rats in each group were used for this assay, and each sample was run in duplicate. The prefrontal cortex was weighed and homogenised at 1 g/10 mL in phosphate buffer solution with 1 mM phenylmethylsulfonyl fluoride (PMSF) and 1 mM ethylene glycol-bis(β-aminoethyl ether)-N,N,N',N'-tetraacetic acid (EGTA). The microtiter plates (96-well flat-bottom) were gently shaken for 60 min at 37 °C with 40 μL samples diluted or 50 μL standards at concentrations of 75–1,200 ng/L 5-HT and 62.5–1,000 ng/L dopamine, 10 μL of anti-5-HT or anti-dopamine monoclonal antibody and 50 μL streptavidin-enzyme. The plates were washed 5 times with wash solution, chromogenic reagents A and B were added in turn for 10 min at 37 °C, and the stop solution was added. The hormone levels were determined in a microplate reader at an absorbance of 450 nm. The standard curve demonstrated a direct relationship between optical density and 5-HT or dopamine concentration.

### Statistical analysis

SPSS 16.0 was used for data analysis. A one-way analysis of variance (ANOVA) followed by the Student–Newman–Keuls test for multiple comparisons were used to compare differences among groups. Student’s t-tests were used when two groups were compared. The relationships between total anxiety and total depression scores were examined with Pearson’s correlation coefficient analysis. Data are presented as means ± standard error. *P* < 0.05 was considered significant.

## Results

### Facial withdrawal threshold

**Fig. 1:**
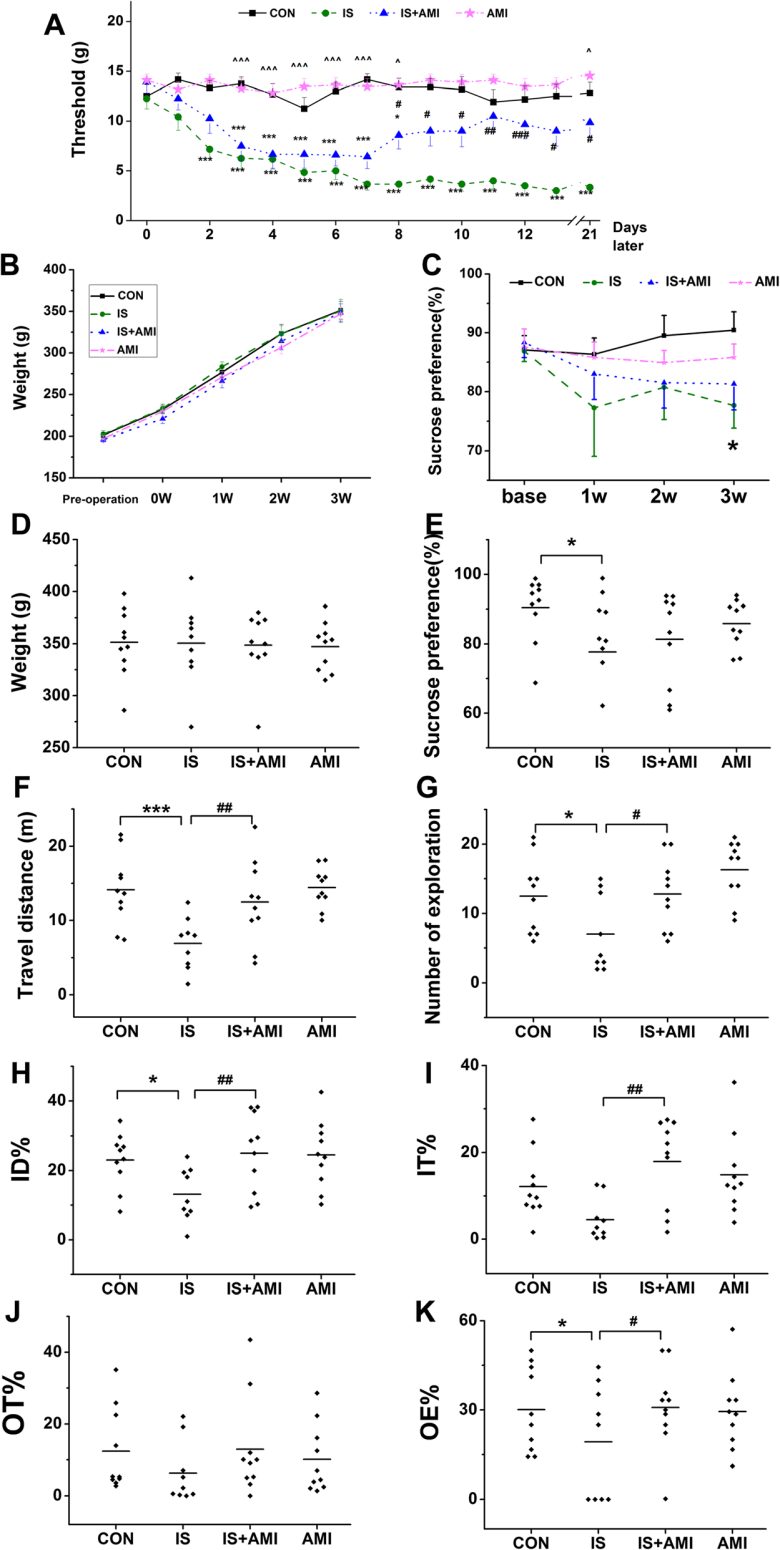
Behavioural studies of allodynia, depression, and anxiety. **a**: Facial withdraw threshold. **b**–**e**: Depressive behaviours. **f**–**g**: Anxiety behaviours. **a**–**c**: The horizontal axis shows time, and the vertical axis shows the values. The facial withdraw threshold (**a**) decreased gradually in the inflammatory soup (IS) and IS + amitriptyline (AMI) groups, and was significantly different form the control (CON) group on Day 3 (***P < 0.001, IS and IS + AMI group vs. CON group). However, the threshold increased gradually in the IS + AMI group on Day 8 and was significantly different from the IS group (#*P* < 0.05, vs. IS group). No difference in weight was detected among the four groups (**b**, **d**) (*P* > 0.05). Sucrose preference (**c**, **e**) was significantly lower in the IS group than that in the CON group after 21 d of administration (*P < 0.05, IS vs. CON); no difference was found between the IS and IS + AMI groups. Travel distances (**f**), number of explorations (**g**), and inner zone distance percent (ID%) (**h**) on the open field (OF) test were all significantly lower in the IS group than those in the CON group but recovered after AMI treatment (*P < 0.05 and ***P < 0.001, IS vs. CON; #*P* < 0.05 and ##*P* < 0.01, IS vs. IS + AMI). Inner zone time percentage (**i**) tended to be lower in the IS group. Open-arm time percentage (OT%) (**j**) and entry percentage (OE%) (**k**) on the elevated plus maze test were lower in the IS group than those in the CON group; only OE% was significantly different (*P < 0.05, IS vs. CON; #*P* < 0.05, IS vs. IS + AMI)

Facial withdrawal threshold was measured the day before administration and everyday just before administration. No significant differences (*P* > 0.05, one-way ANOVA) were detected among the four groups on the first day. After administration, the thresholds decreased significantly in the IS and IS + AMI groups (*P* < 0.001 vs. CON group). Thresholds in the IS group held steady at 2 g after 1 week of administration. However, the thresholds in the IS + AMI group increased gradually and were significantly different on Day 8 (*P* < 0.05, IS vs. IS + AMI). A significant difference was detected between the IS + AMI and the AMI groups during the late half of the first week (*P* < 0.001, IS + AMI vs. AMI) and on Day 21 (*P* < 0.05, IS + AMI vs. AMI) (Fig. 1a).

### Depression tests

Body weight, sucrose preference, travel distance, and number of explorations in the OF test were used to evaluate depression. Weight increased as time went on; however, no differences were found among the four groups (*P* > 0.05) (Fig. 1b, d). Sucrose preference, which reflects anhedonia, is a classical method to evaluate depression. Sucrose preference decreased in the IS group during the 21 d. A significant difference was detected between the IS and CON groups after 21 d (*P* < 0.05, IS vs. CON) (Fig. 1c, e). Locomotor and rearing behaviours can also reflect depression. The distance travelled by rats in the IS group was significantly less (*P* < 0.001, IS vs. CON) (Fig. 1f), and they explored less area (*P* < 0.05, IS vs. CON) (Fig. 1g) than rats in the CON group. AMI significantly improved locomotor (*P* < 0.01, IS vs. IS + AMI) and rearing behaviours in the IS group (*P* < 0.05, IS vs. IS + AMI), but not sucrose preference (*P* > 0.05). Administering AMI only did not affect the results (*P* > 0.05).

### Anxiety tests

The ID% and IT% value on the OF test and OT% and OE% on the EPM test were used to evaluate anxiety. Anxious rats are afraid to explore and will stand in a safer place, such as the outer perimeter of the OF or the closed arm of the EPM. ID% and OE% were significantly lower in the IS group than those in the CON group (*P* < 0.05, IS vs. CON) (Fig. 1h, k). However, no differences were detected in the IT% or OT% between the CON and IS groups (Fig. 1i, j). AMI significantly improved ID% (*P* < 0.01, IS vs. IS + AMI), IT% (*P* < 0.01, IS vs. IS + AMI), and OE% (*P* < 0.05, IS vs. IS + AMI).

### Depression and anxiety scales

Tables 1 and 2 show the depression and anxiety scales for rats, respectively. Fewer points indicate more depression or anxiety. Depression can lead to overweight and underweight [16]. A full score on the new scale was 5 points. Weight of 330–360 g (normal weight for 9–10-week-old rats) = 5. An increase or decrease of 10 g was –1 point for each 10 g increment. A score of 0 was given for weights <300 g or >390 g. On the sucrose preference test, a full score of 10 points was given to >95% sucrose preference; <50% was 0. Each 5% increase was 1 point. On the OF test, locomotor behaviour > 20 m was 5 points, no movement was 0; each 5-m increase was 1 point. A full score for rearing behaviour was also 5 points, >20 times was 5 points, no exploration was 0; every 5× increase was 1 point (Table 1).

**Table 1:** Scale of depression in rats

Depression indexes	Interval			Scores
Weight (g)		<300		1
	300 ≤		<310	2
	310 ≤		<320	3
	320 ≤		<330	4
	330 ≤		<340	5
	340 ≤		<350	5
	350 ≤		<360	5
	360 ≤		<370	4
	370 ≤		<380	3
	380 ≤		<390	2
		390≤		1
Sucrose preference (%)	50 ≤		<55	1
	55 ≤		<60	2
	60 ≤		<65	3
	65 ≤		<70	4
	70 ≤		<75	5
	75 ≤		<80	6
	80 ≤		<85	7
	85 ≤		<90	8
	90 ≤		<95	9
		95≤		10
Travel distance (m)		0		0
	0 <		<5	1
	5 ≤		<10	2
	10 ≤		<15	3
	15 ≤		<20	4
		20≤		5
Explpration number		0		0
	0 <		<5	1
	5 ≤		<10	2
	10 ≤		<15	3
	15 ≤		<20	4
		20≤		5

**Table 2:** Scale of anxiety in rats

Anxiety indexes	Interval			Points
Inner zone distance percent, ID%	0 ≤		<1	0
	1 ≤		<5	1
	5 ≤		<10	2
	10 ≤		<15	3
	15 ≤		<20	4
	20 ≤		<25	5
	25 ≤		<30	6
		30≤		7
Inner zone time percent, IT (%)	0 ≤		<1	0
	1 ≤		<5	1
	5 ≤		<10	2
	10 ≤		<15	3
	15 ≤		<20	4
	20 ≤		<25	5
	25 ≤		<30	6
		30≤		7
Open arm time percent, OT (%)	0 ≤		<1	0
	1 ≤		<5	1
	5 ≤		<10	2
	10 ≤		<15	3
	15 ≤		<20	4
	20 ≤		<25	5
	25 ≤		<30	6
		30≤		7
Open arm entries percent, OE (%)	0 ≤		<1	0
	1 ≤		<20	1
	20 ≤		<25	2
	25 ≤		<30	3
	30 ≤		<35	4
	35 ≤		<40	5
	40 ≤		<45	6
		45≤		7

A full score for the anxiety scale was 7 points. More than 30% for ID%, IT%, or OT% was scored as 7 points, and <1% was 0. Every 5% increase was 1 point. More than 45% for the OE% was a full score of 7, <1% was 0, and 1–20% was 1 point. Every 5% increase was 1 point (Table 2).

**Fig. 2:**
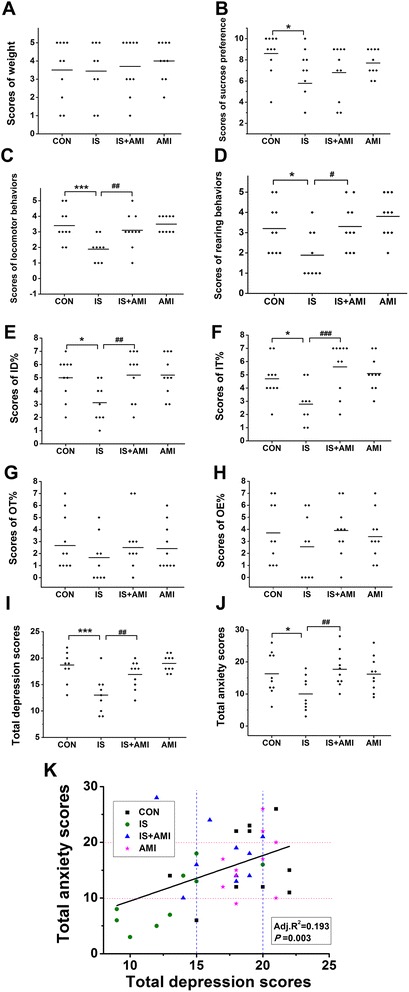
Depression and anxiety scale scores. **a**–**d**: Depression-associated scores. **e**–**h**: Anxiety-associated scores. Facial withdraw threshold. **b**–**e**: Depressive behaviours. **i**: All depression scores together. **j**: All anxiety scores together. **k**: Relationship between total anxiety and total depression scores. The results were similar to the primary behavioural data, except for the inner zone time percentage (IT%) (**f**), which was significantly different, and open-arm entries (OE%) (**h**), in which the significant difference disappeared. Total depression (**i**) and anxiety scores (**j**) were significantly lower in the IS group (*P < 0.05, ***P < 0.001, IS vs. CON) but improved in the IS + AMI group (##*P* < 0.01, IS vs. IS + AMI). More depression paralleled more anxiety in most of the rats (**k**). However, a few rats had only anxiety or only depression

The results were similar to the primary behavioural data (Fig. 2), except IT% (Fig. 2f), which was significantly different, and open-arm entries (Fig. 2h), in which the significant difference disappeared.

Finally, we added all of the scores together. As a result, most of the IS group rats were more depressed and anxious than those in the CON group. However, not all control rats had normal emotions, and not all IS group rats were depressed or anxious. AMI was not only useful for depression, but also anxiety, if used very early in the onset of headache (Fig. 2i, j). We tested the relationship between anxiety and depression, and more depression usually indicated more anxiety. However, a few rats were only anxious or depressed (Fig. 2k).

### Concentration of 5-HT and dopamine in the prefrontal cortex

**Fig 3:**
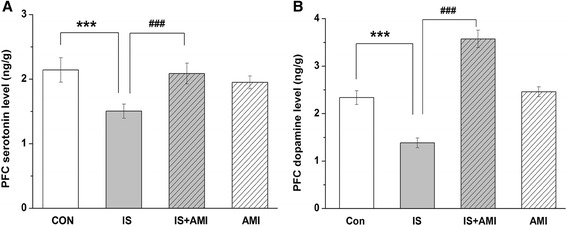
Concentrations of serotonin (**a**) and dopamine (**b**) in the prefrontal cortex measured by ELISA. 5-HT and dopamine concentrations were significantly lower in the IS group than those in the CON and IS + AMI groups in the prefrontal cortex. No differences were found between the CON and AMI groups (***P < 0.001, IS vs. CON; ###*P* < 0.001, groups IS vs. IS + AMI.)

5-HT and dopamine concentrations in the prefrontal cortex decreased consistently in the IS group. Repeated administration of AMI did not increase 5-HT levels; however, when AMI was used to treat pain, 5-HT and dopamine levels increased significantly (Fig. 3).

## Discussion

The present study investigated whether IS leads to depression and anxiety and whether a small dose of AMI can effectively treat depression and anxiety. The results showed that IS group rats had cutaneous allodynia and were depressed and anxious. AMI was able to reverse the allodynia and decreased the occurrence of depression and anxiety.

### Affective disorders

Many studies have demonstrated a relationship between migraine and mental disorders. Cohort and epidemiological studies have shown that migraineurs have a higher risk of depression compared with those having no history of headache without exception [23–25] and compared with patients having other severe headache types [26]. This indicates that headache is not the only cause of depression but also the migraine itself [26]. Ratcliffe first reported a large population-based sample investigating physician-diagnosed migraine. The results showed that a past-year migraine was significantly and positively associated with depression, dysthymia, bipolar disorder, panic attacks, panic disorder, agoraphobia, and simple phobia [adjusted odds ratios, 1.74–3.21] [5]. Anxiety differs from depression, but clinical and epidemiological surveys have confirmed that both disorders commonly occur together [27, 28]: 42.3–84.6% of patients with migraine suffer from depression and anxiety, and 66.1–85.7% of patients with anxiety suffer from migraine and depression [3]. All of these studies showed a correlation among migraine, depression, and anxiety in different races and cultural backgrounds using different research methods.

Few animal studies have evaluated the relationship between migraine and mental disorders, possibly because animal models that recapitulate the pathophysiology of migraine are scarce. In this study, we used an accepted chronic migraine IS animal model. IS was administered on the dura matter and caused an instant transient reduction in the pain threshold that lasted approximately 3-6 h. Chronic IS exposure for more than 1 week can lead to a persistently reduced pain threshold [13]. This animal model is reproducible [11]; we used it to demonstrate the relationship between migraine and affective disorders.

Our results showed that rats with chronic migraine were more depressed and showed particularly less locomotor behaviour. Sucrose preference and rearing behaviours were also significantly different compared with those of the control rats. The rats with chronic migraine were more anxious, as demonstrated by in the IT% and OE% but not the time percentages of the two tests. In addition, concentrations of 5-HT and dopamine in the prefrontal cortex were significantly lower in the IS group than those in the control group. These results are consistent with clinical studies, indicating that this chronic migraine animal model fits the phenotype and can be used to elucidate the molecular mechanism of depression due to chronic migraine. The forced swim test is widely used to assess depression and antidepressant activity. However, we did not use this test because it can lead to depression and may affect the results of other tests.

The scales developed for rats in the present study showed that depression and anxiety were positively correlated. Depression and anxiety are two classes of psychiatric disease. Major depressive disorder is a mood disorder that can be coupled with mania, whereas anxiety is a normal physiological phenomenon; too much anxiety leads to generalised anxiety disorder or panic disorder. Clinical and epidemiological studies have reported that depression and anxiety often co-occur. More than 75% of patients with a depressive disorder also suffer from an anxiety disorder [29]. These comorbidities lead to severe symptoms and increase suicide risk [30]. Most of the rats with chronic headache in the present study suffered from depression and anxiety. A linear relationship was found between the total depression score and the total anxiety score. Rats with a total depression score < 15 points also had <10 points on the total anxiety score, and they were always IS group rats. Rats whose total depression score was >20 points also scored >10 points on the total anxiety score, and they were always from the CON and AMI groups. The comorbidity ratio in these rats was similar to that of patients, which verified the rational and feasibility of our scales.

The risk factors for comorbid depression and anxiety may be related with headache frequency and sex distribution. We plan to change the dosage and frequency of IS and use different sexes of rats to explore the aetiology of this comorbidity.

### Effect of low-dosage AMI

AMI is a tricyclic antidepressant that has been widely used for half a century. It has not only been used to treat depression but also to relieve chronic pain, including diabetic peripheral neuropathy, post herpetic neuralgia, fibromyalgia, and migraine. Early research suggested that AMI blocks reuptake of 5-HT and norepinephrine (NE) in the synaptic cleft, which effectively increases their concentrations. The onset time was about 1 month, and the dosage used was 100–300 mg/d. AMI also acts quicker on ion channels by blocking both tetrodotoxin-sensitive (TTX-s) and TTX-resistant (TTX-R) Na + currents in the dorsal root ganglion [31]. It also blocks TTX-s Nav1.4 current in gene-transfected HEK293T [32] and TTX-R Na + channels in trigeminal ganglion neurons (TG) [33]. Our team reported previously that AMI blocks the TTX-R Nav1.8 and TTX-R Nav1.9 current in TG [34, 35].

In the present study, 5-HT concentration in the cortex did not increase after 21 d of AMI administration alone, perhaps because the effect of blocking reuptake didn’t occur within 3 weeks. However, 5-HT and DA concentrations did increase when AMI and IS were co-administered compared with IS alone. This result may be due to an indirect effect of relieving pain first, and then reducing depression and increasing 5-HT and DA concentrations. Next, we wanted to demonstrate and observe nociceptive behaviour immediately after AMI administration. However, the rats were sleepy and were less active after being given the AMI; therefore, it was very difficult to distinguish whether the decrease in nociceptive behaviour was caused by being pain-free or sleepy.

### Depression and anxiety disorder scales

Many indices have been used to evaluate affective disorders in animals; however, some studies have shown that some of these indices are significantly different, whereas others are not. All kinds of scales are used to evaluate disorders clinically and can also be used to evaluate animals. The most interesting finding of the present study was that not all IS rats developed depression or anxiety. All data mean something, even outliers. Therefore, a future study is necessary to find out the reason.

### Limitations

The present study has some limitations. First, female animals didn’t contain in this study, but it may relate to migraine and affective disorders, so female rats would be added in future studies. Second, the scales was not wide application and needed to be confirmed. Lastly, the present study only found out the behaviors and the mechanism need to be study in the future.

## Conclusions

The present study evaluated the depression and anxiety behaviors on a chronic migraine animal model and the therapeutic effects of low-dose amitriptyline. Most of the chronic migraine rats were present depression and anxiety like behaviors, but not all. We used new scales of depression and anxiety to comprehensively assess and wanted to find out the differences between depression and non-depression rats in the chronic migraine in the future study.

## Abbreviations

5-HT: serotonin
AMI: amitriptyline
CON: control
EPM: elevated plus maze
ID%: inner zone distance percent
IS: inflammatory soup
IT%: inner zone time percent
OE%: open arm entries percent
OF: open field
OT%: open arm time percent
